# DisclosuR: Advancing firm communication analysis through an innovative R package for enhanced textual insights

**DOI:** 10.1016/j.mex.2024.102909

**Published:** 2024-08-13

**Authors:** Jonas Röttger, Rick Aalbers

**Affiliations:** Radboud University, Department of Business Administration, Heyendaalseweg 141, 6525 AJ Nijmegen, the Netherlands

**Keywords:** Firm communication, LexisNexis, Impression management, Natural language processing, Structured Analysis of Corporate Communications in R

## Abstract

Firm and executive written communication allows researchers to explore firm strategy and executive personality. Two data sources have received increased interest in this matter: firm press releases and earnings call transcripts. However, while researchers can obtain these data sources through services like LexisNexis, they often come in unstructured formats that do not directly allow fine-grained quantitative analysis through statistical software. To address this challenge, we developed *disclosuR*, an innovative R package that transforms unstructured PDF press releases and earnings call transcripts into structured data frames, facilitating advanced text analysis. disclosuR stands out by providing unique features such as speaker-level language analysis and identifying temporal communication patterns within press releases. These functionalities empower researchers to conduct granular and reproducible quantitative analyses, significantly advancing the management literature. By enabling the seamless integration of text data into R, our package not only enhances the reproducibility of social science research but also opens new avenues for examining executive communication dynamics and strategic firm disclosures.

• Convert LexisNexis PDFs to structured R data frames
• Standardize text analysis of firm communication

## Graphical abstract

**Figure:**
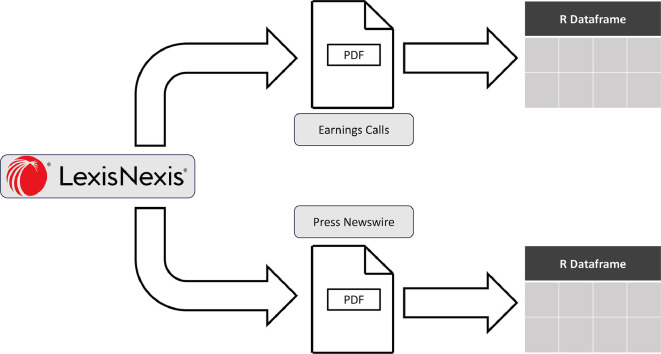

Specifications table

**Table:** 

Subject area:	Business, Management and Accounting
More specific subject area:	Text analytics for press releases and earnings calls from LexisNexis
Name of your method:	Structured Analysis of Corporate Communications in R
Name and reference of original method:	N.A.
Resource availability:	Related R code is available in: https://github.com/jonasroettger/disclosuR https://cran.r-project.org/web/packages/disclosuR/index.html

## Background

Researchers have increasingly turned to firm disclosure as a valuable resource for analyzing firms' strategic communication [1]. By examining the language and content of these public statements, researchers have identified patterns and motives behind various corporate actions, such as acquisitions and leadership changes. For example, press releases have been used to uncover the impression management tactics employed by firms in their acquisition announcements, shedding light on the motivations behind these strategic moves [2]. Similarly, press releases have been analyzed to understand how firms communicate changes in leadership, providing insight into the importance of leadership transitions and how firms seek to manage stakeholder perceptions of these events. Overall, using press releases as a data source for understanding corporate communication has proven to be a valuable tool for researchers seeking to better understand the strategic decisions and actions of firms in today's complex business environment.

As part of this development, the burgeoning field of textual analytics has recently seen an uptick in interest, particularly from management scholars exploring company news and executive communication for insights into market dynamics and leadership characteristics. This shift towards text data underscores its value across disciplines like finance, management, and accounting, where it serves as a critical resource for both granular and broad-scale research inquiries [3,4].

**Fig. 1:**
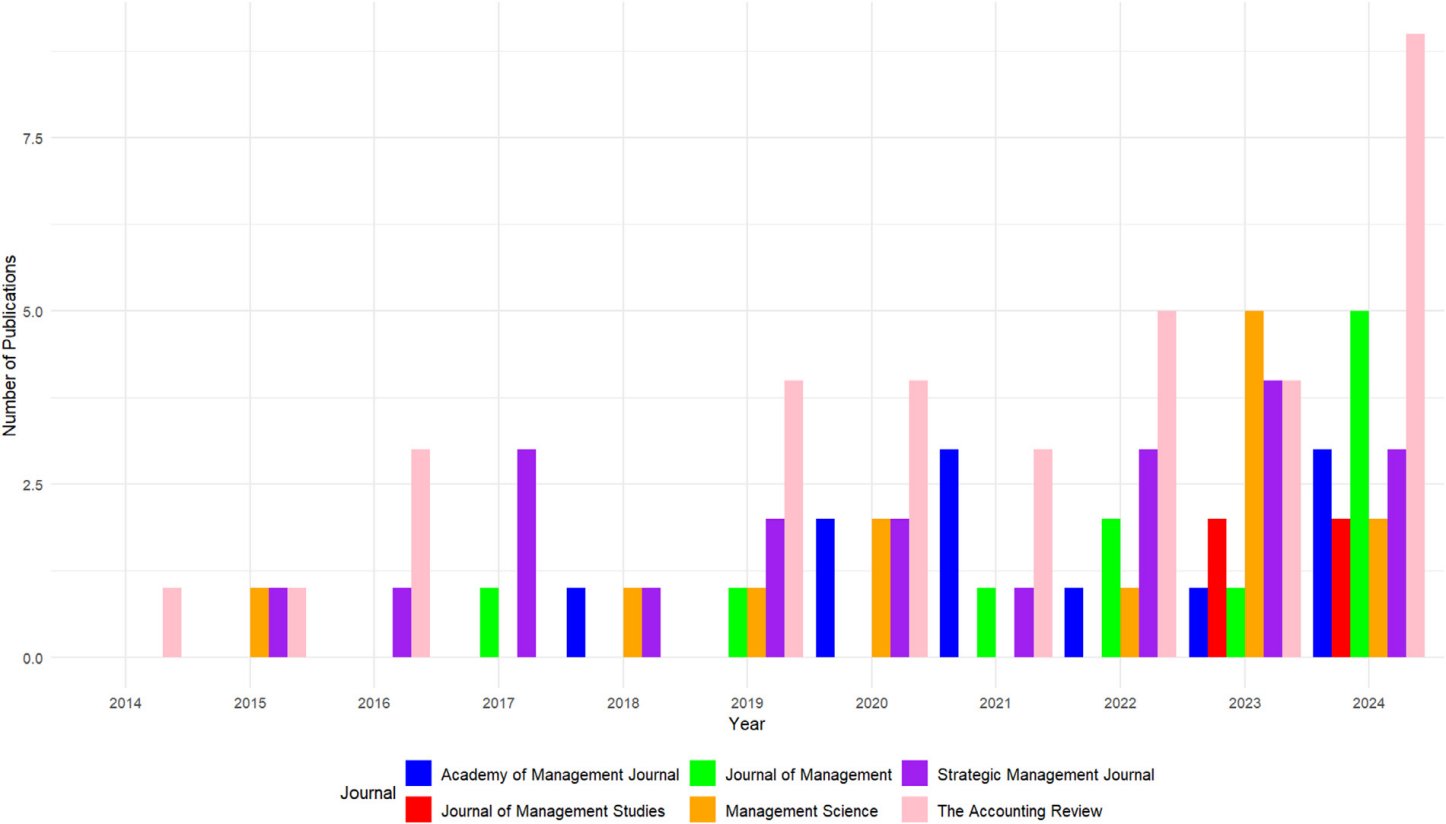
Number of publications using earnings calls as a data source.

Despite its proven utility, the full potential of text data, however, remains untapped due to a lack of domain-specific analytical tools, which stymies result reproducibility and the establishment of analytical standards within the management field. The increasing inclination towards programming for statistical text analysis presents opportunities for bespoke data examination. Such programmatic methods permit the handling of voluminous datasets that are unfeasible for manual analysis, but they also introduce variability in the preprocessing phase that can obfuscate data comparisons and impede reproducibility. For instance, while Fig. 1 demonstrates the current trend toward using earnings calls as a data source in management journals, only a few articles report on the selection of text from larger transcripts and how they analyzed individual data.

Addressing these challenges, this paper introduces the R package disclosuR, designed to standardize the analysis of press releases and executive communications. This tool ensures analytical consistency and fosters comparability across studies by streamlining data preprocessing—a crucial step in textual analysis. DisclosuR facilitates the conversion of documents such as earnings call transcripts into structured data frames, enabling detailed analysis, including speaker identification and sentiment assessment. By providing tools for rigorous textual analysis, disclosuR supports the exploration of strategic inclinations and personality traits among corporate leaders. The package standardizes oftentimes unreported preparation and processing steps of text data in business and management science. Its contribution is valuable for increasing the replicability of results and enhancing the comparability of findings.

For instance, when analyzing earnings calls disclosuR assigns each speaker an individual ID and each quote a timestamp, allowing for the analysis of role-specific communication and trends in language, such as the sentiment distribution over a document by a specific speaker.

Through practical applications in strategic management research, disclosuR's utility is demonstrated in developing metrics for CEO language scriptedness and impression management in corporate communications. These examples underscore the package's contribution to research efficiency and reproducibility. By enhancing process transparency, disclosuR lowers barriers for subsequent research, aligning with broader calls for increased reproducibility in social science. Moreover, it simplifies data preparation, allowing researchers to allocate more resources to substantive analysis rather than preliminary data management.

## Method details

### Data collection

Press releases and earnings call transcripts are available through various data providers, like LexisNexis, Factiva, or Seeking Alpha [5]. We draw on LexisNexis for our package development because it is widely available at educational institutions. LexisNexis is an online platform that provides access to a vast collection of news wire data, including press releases, news articles, and transcripts of conference calls conducted by publicly traded firms. This resource can be precious for researchers interested in studying how firms communicate with their stakeholders and the public, as it allows for analyzing both the content and linguistic patterns of these communications. Moreover, this data contains text spoken by individual executives, often regarded as a source of self-disclosure that can be used to proxy personality traits [6]. While LexisNexis provides good coverage of large and Western events, applying it in other context might induce sample selection bias. Using LexisNexis, researchers can search for news articles and press releases related to a specific firm, industry, or topic of interest. They can also retrieve transcripts of conference calls, which typically include the remarks made by top executives and managers of the firm during the call, as well as the questions and answers from analysts and investors. The data available on LexisNexis can be used to analyze a firm's communication strategy over time, identifying patterns and changes in how the firm communicates with different stakeholders, such as customers, employees, investors, and the media. Researchers can also use this data to investigate the impact of specific events, such as mergers and acquisitions, on a firm's communication strategy and relationship with stakeholders. LexisNexis is organized in a way that enables researchers to use keyword-based prompts to search for news items while also applying advanced filters on the period and outlets to consider. For instance, numerous researchers have relied on the FairDisclosure outlet to obtain conference call transcripts, like earnings calls. Other scholars have turned to the prominent newswires of Business Newswire and PR Newswire to study organizational impression management patterns. We will provide examples of the usage of disclosuR in pre-processing of both data types (i.e., earnings call transcripts and newswire texts) and demonstrate how researchers could build on these to tackle new research questions.

### Data preparation

#### Conference call analysis

**Fig. 2:**
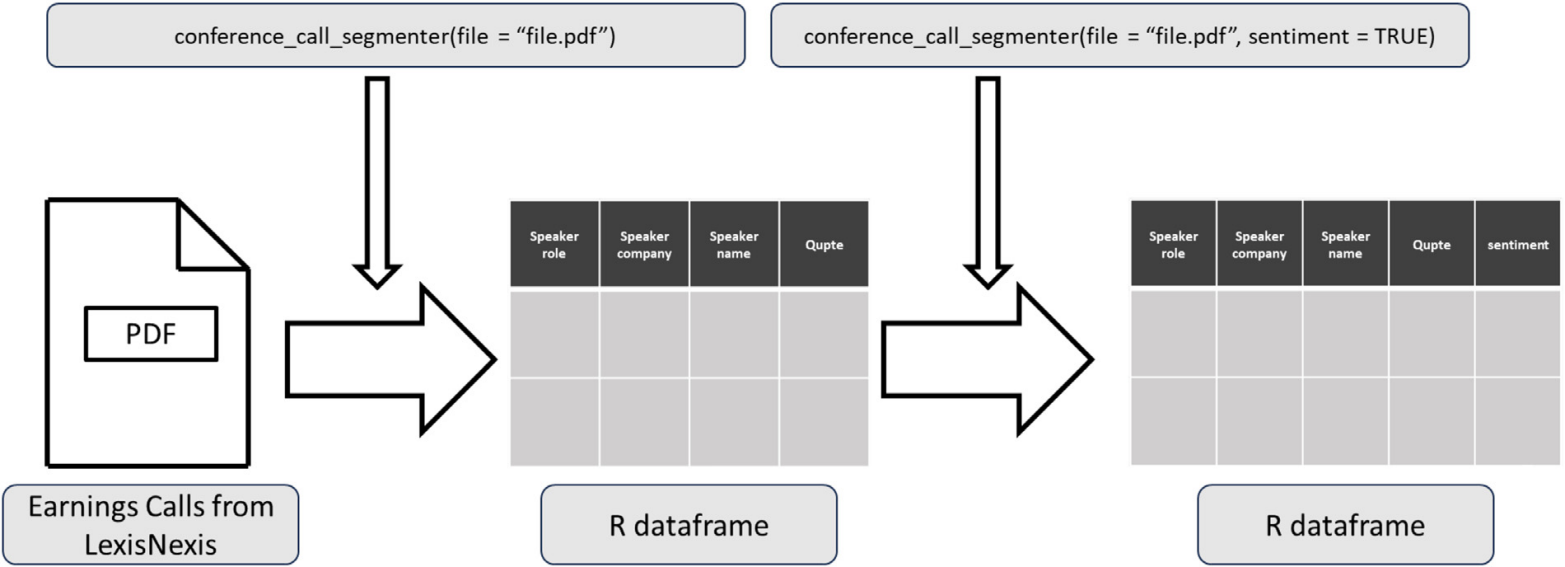
Workflow of the conference_call_segmenter function.

The FairDisclosure outlet provides access to a wealth of corporate earnings call transcripts in PDF format. However, analyzing these transcripts can be daunting, especially for researchers who need to extract specific information from large volumes of earnings calls. The *disclosuR* package offers a solution in the form of the *conference_call_segmenter* function. This powerful tool can convert PDF transcripts into an R data frame, assigning each quote its own row as a text column. In addition to the text, the function provides information on the speaker's name, role, company, call section (presentation or Q&A), and relative position in the call. Fig. 2 illustrates the workflow of the conference_call_segmenter function.

With this data frame at their disposal, researchers can perform various text analysis techniques to gain insights into corporate communication strategies and financial performance. Moreover, the *conference_call_segmenter* function provides additional arguments that researchers can set to TRUE to apply additional analysis to the segmented text. For instance, by setting the argument sentiment to TRUE, researchers will receive the sentiment of each quote calculated based on the *SentimentAnalysis* package [7] which applies a dictionary-grounded measure based on three popular dictionaries: (1) the Harvard-IV dictionary, (2) Henry's Financial dictionary [8], and (3) the Loughran-McDonald Financial dictionary [9]. This functionality is of relevance to data scientists in the strategy domain as it enables them to perform text analysis techniques on a given data frame in order to gain insights into corporate communication strategies and financial performance. The availability of the *conference_call_segmenter* function with additional argument options hence allows researchers to apply further analysis to the segmented text, enhancing their ability to extract valuable information from the data. The results will be structured similar to those presented in Table 1.

**Table 1:** Export the first line of a segmented conference call (only one row displayed) created by the conference_call_segmenter function.

Variable_name	Quote_01	Quote_02
year	2015	2015
quarter	Q4	Q4
date	2015-01-20	2015-01-20
weekday	Tuesday	Tuesday
section	presentation	presentation
speaker_name	OPERATOR	Sarah business
speaker_role	OPERATOR	CEO
speaker_company	OPERATOR	Company X
host_company	0	1
host_CEO	0	0
host_CFO	0	0
host_Chairman	0	0
quote_index	1	1
quote	Good day, ladies and gentlemen. Thank you for sta	Thank you, and good morning from my side

Note. Characters variables are cut off at 50 characters.

#### Press release analysis

To systematically convert unstructured newswire data to structured R data frames, we developed the *newswire_segmenter* function. The *newswire_segmenter* function is applied to a press release to extract information and perform various analyses of the text data. The function takes a PDF document containing the news-wire document as input and transforms it into an R data frame consisting of one row per newswire article. The function has several optional parameters that allow for additional analyses, such as sentiment analysis, emotion analysis, regulatory focus analysis, laughter analysis, narcissism analysis, and text clustering. The function first reads the PDF document and preprocesses the text by removing line breaks and extra whitespace. Next, it identifies the newswire from a pre-defined list based on patterns found in the text. The title, newswire, date, and text of the press release are extracted from the document and added to the data frame. Optional analyses are then performed based on the specified parameters. If sentiment analysis is enabled, the function uses the *analyzeSentiment* function to calculate sentiment scores for each quote in the press release text [7]. Emotion analysis uses the *get_nrc_sentiment* function [10]. Regulatory focus analysis calculates the number of words indicative of promotion and prevention focus based on a pre-defined dictionary [11]. Laughter analysis counts the number of times laughter is indicated in a quote. Narcissism analysis counts the pronoun usage and calculates the ratio of first-person singular to first-person plural pronouns [12]. Text clustering applies document categorization using a dictionary developed by Graffin et al. (2016) based on keywords found in the preprocessed text. Finally, the function assigns a valence category to each press release based on the valence to topic allocation developed by prior research [2]. Terms associated with positive, negative, neutral, and ambiguous categories are matched against the *category_Graffin* column, and the *valence_category* is determined accordingly. The results will be structured similar to Table 2.

**Table 2:** Categorized newswire data (only one row displayed) created by the newswire_segmenter function.

Variables	example_newswire.pdf
title	Accenture Acquires SolutionsIQ, Adds Leading Agile Transformation Expertise and Services; Acquisitio
newswire	Business Wire
date	17319
text	Accenture Acquires SolutionsIQ, Adds Leading Agile Transformation Expertise and Services; Acquisitio
weekday	Friday
WordCount	858
SentimentGI	0.1410256
NegativityGI	0.1107226
PositivityGI	0.2517483
SentimentHE	0.02214452
NegativityHE	0.01748252
PositivityHE	0.03962704
SentimentLM	0
NegativityLM	0.06060606
PositivityLM	0.06060606
RatioUncertaintyLM	0.04312354
SentimentQDAP	0.1048951
NegativityQDAP	0.06526807
PositivityQDAP	0.1701632
preprocessed_title	accenture acquires solutionsiq adds leading agile transformation expertise services acquisition exp
Earnings releases	0
Earnings guidance	0
Change in dividend rate	0
New product	0
Customer win	0
Social good (e.g., donation, sponsorship), training, professional development	0
Received award from third party	0
Buyback or split stock	0
Results of a sponsored study	0
New executive or director	0
Divestiture or plant closing	0
Settlement of litigation or other legal dispute	0
Executive retirement	0
Change of stock exchange listing	0
Debt issuance	0
Other acquisition	1
Completion of another acquisition	0
Recall or safety issue	0
Partnership announcements	0
Other	0
category_Graffin	Other acquisition
valence_category	negative

## Method validation

### Two practical examples: Language scriptedness and impression offsetting

To demonstrate the usefulness of the disclosuR package, we will describe two practical examples of text analysis that included disclosuR functions as part of the data preparation and analysis. In the first example (Example 1), we will train a random forest classifier on CEO speech in earnings calls to create a measurement for language scriptedness. In the second example (Example 2), we will apply our self-developed impression management dictionary to a body of firm press releases to investigate whether firms use impression offsetting around acquisition announcements.

Example 1 Analyzing CEO speech - Developing a binary indicator for language scriptedness

Earnings calls are composed of two parts. The first contains a presentation by the management, whereas the second part covers the Q&A session during which security analysts can inquire about the management of the firm's strategy and results [13]. Scholars have drawn on either of these parts depending on the research goal. For instance, scholars interested in firm impression management might use the presentation section since it indicates a voluntary communication tactic and reflects the firms’ attempts to steer outsider perceptions. In contrast, scholars interested in gauging CEO personality often use the Q&A session because it contains the unprepared part of the CEO speech, which is more indicative of CEO personality [3].

We leverage the earnings calls’ inherent structure and train two machine-learning algorithms to differentiate between CEO scripted speech used during the presentation section and CEO free speech used during the Q&A session. Language scriptedness is the degree to which a communication is pre-planned or follows a predetermined script, often resulting in language that sounds rehearsed or formulaic. This degree of rehearsal can, for instance, indicate more deceptive speakers who try to nudge stakeholders into contentment [14]. Since this task requires a binary classification, we used a random forest algorithm [15]. Random Forest is a machine learning algorithm that creates an ensemble of decision trees and combines their predictions to improve the accuracy and robustness of the model. Respectively, decision trees are a machine learning algorithm that recursively splits the data into subsets based on the most important features to create a hierarchical set of rules for predicting the target variable. Random forest classifiers are increasingly used in strategic management research when creating categorical variables in a large dataset is challenging because manual coding is not feasible [16].

We first downloaded all earnings calls from LexisNexis that were available from FairDisclosure for all S&P500 companies between 2012 and 2021. Subsequently, we used the function conference_call_segmenter_folder from the disclosuR package to load all PDFs into R as one data frame. This more high-level function iteratively applies the conference_call_segmenter function to all PDF files in the target folder. Using the host_CEO binary variable, we filtered the data to only contain quotes from the host company's CEO. Subsequently, we followed standard procedures for text preparation in natural language processing. Firstly, all numerical information and punctuation are removed. Secondly, all stop words were eliminated from the text. Thirdly, all words were converted to all-lowercase. Finally, all words were stemmed, which refers to the process of reducing a word to its base or root form to improve the efficiency and accuracy of natural language processing algorithms. Stemming uses the wordStem function from the SnowballC package [17]. Subsequently, the document term matrix was created using the DocumentTermMatrix function from the tm package [18]. This matrix contains the word frequencies across all the documents. The rows correspond to the documents, and the columns correspond to words in those reviews. The values in the matrix are the frequency of the word across the document. The document term matrix may have many zeroes in its cells, which creates sparsity. To reduce sparsity, we removed words with many zeroes across the documents using the removeSparseTerms function.

Before building a predictive model, we needed to convert the document term matrix into a data frame. Furthermore, we made the variable names R-friendly and added the dependent variable to the data set, meaning we added the section indicator (i.e., presentation versus Q&A section).

## Training a random forest model

Following common practice in machine learning (e.g., [19]), we first split our data into a training and test data set, using a 70/30 ratio. Subsequently, we trained a random forest model on our training data using the randomForest function from the eponymous R package RColorBrewer [20]. It is important to consider the number of variables (i.e., words) that went into the initial training of the random forest model and save the corresponding document term matrix. This matrix can then limit future text bodies to the words used in the training phase since, otherwise, the model cannot predict [21].

## Results

We applied the random forest classifier to our test dataset, and the resulting confusion matrix showed that the model predicted presentation and Q&A sessions with an accuracy of 96.2 %. Specifically, out of the 6000 sessions in the test dataset, the model correctly classified 2818 presentation sessions and 2955 Q&A sessions. The model misclassified 45 presentation sessions as Q&A sessions and 182 Q&A sessions as presentation sessions. Overall, the random forest classifier showed high accuracy in predicting the type of session, with a precision of 98.4 % and a recall of 93.9 % for presentation sessions and a precision of 94.2 % and a recall of 98.5 % for Q&A sessions. These results suggest that the random forest classifier effectively distinguishes between presentation and Q&A sessions in our dataset. Therefore, the trained model can be used to analyze whether a CEO speech in new contexts, e.g., TV interviews, is scripted.

Example 2 Measuring impression offsetting in acquisition announcements

Various studies in anticipatory impression management (AIM) demonstrated that firms alter their communication paper around events to which they anticipate a negative market reaction [2]. While the AIM literature covered various tactics, in this example, we will focus on impression offsetting, defined as amplifying positive communications to mitigate potential negative consequences or reactions to an announcement likely to be perceived as negative [22]. We drew on an established framework that categorizes press releases into positive, neutral, or negative based on the topic they cover [2]. This dictionary is implemented into the disclosuR package, and the topics above are assigned using the frequency of pre-defined keywords.

Applying the categorization to a set of S&P 500 acquisition announcements

To see how well our measurement performs, we collected a set of acquisition announcements by S&P 500 companies between 2010 and 2019. Acquisition announcements refer to public statements made by a corporation indicating its intent to acquire another business entity [23]. These disclosures usually detail essential aspects of the intended acquisition, such as the proposed terms of the deal, the companies involved, and the expected timeline for completion [1]. However, investor reactions to acquisition announcements are typically mixed, primarily driven by the perceived benefits and risks associated with the proposed deal [24]. Moreover, it's noteworthy that acquisition announcements are frequently accompanied by impression management tactics, a common one being impression offsetting. This strategy involves simultaneously announcing potentially favorable news alongside acquisition news to dilute the negative impact and divert investor attention [2]. By timing the release of less favorable information, companies can manage stakeholders’ overall perception, endeavoring to create a balanced impression that offsets potential concerns arising from the acquisition, thus mitigating any potential adverse reactions from the investor community. We quantified impression offsetting by counting the number of favorable statements about the focal firm, included in a press release authored by the firm, during a three-day window centered around the acquisition announcement (i.e., one day before and after).

## Results

We analyzed the occurrence of positive event announcements by comparing the observed count to a baseline of the average count of announcements over a three-day period from our measure and a prior study. We established the baseline count for positive event announcements over a three-month period beginning four months prior to and ending one month prior to the acquisition event (i.e., day -121 to day -30). During this period, firms had an average count of 0.0897 positive announcements every three days. However, during the impression offsetting window (also three days), we observed that 44.7 % of firms in our sample (1,883 out of 4,212 acquisitions) had at least one positive event announcement. This is significantly higher than the baseline count (t = 14.48, p < .001), indicating that firms released significantly more positive unrelated news around acquisitions (mean = 4.779) than suggested by the baseline (mean = 0.114).

## Discussion

The disclosuR package allows researchers to analyze firm communication data systematically and reproducibly from LexisNexis. The practical examples illustrate how the package can add value by (a) increasing the comparability of studies drawing on the same unstructured data source and (b) allowing for more fine-grained analysis of text data. Researchers using the package can address future research questions such as: (1) Are there notable differences in communication styles between male and female executives in earnings call transcripts? (2) How consistent is the sentiment in communications across different levels of management within a firm?

## Conclusion

DisclosuR, our R package, transforms unstructured PDF press releases and earnings call transcripts from LexisNexis into structured data frames, facilitating advanced text analysis. It offers tools for speaker-level language analysis, sentiment analysis, and temporal communication patterns. This package enhances reproducibility and standardizes data preprocessing, empowering researchers to delve into corporate communication strategies and executive behaviors. Through practical applications, we demonstrated its utility in identifying scripted CEO speech and impression offsetting in acquisition announcements. DisclosuR simplifies text analysis, enabling more efficient and reproducible research in the management field.

## Limitations

The method described can only process text data acquired through LexisNexis. Researchers relying on other sources, e.g., Factiva, will have to adjust the method.

## Ethics statements

Not applicable.

## CRediT authorship contribution statement

**Jonas Röttger:** Conceptualization, Methodology, Software, Validation, Formal analysis, Investigation, Data curation, Writing – original draft, Visualization. **Rick Aalbers:** Resources, Writing – review & editing, Supervision, Project administration, Funding acquisition.

## Declaration of competing interest

The authors declare that they have no known competing financial interests or personal relationships that could have appeared to influence the work reported in this paper.

## Data Availability

The authors do not have permission to share data.
